# Programming Robots by Demonstration Using Augmented Reality

**DOI:** 10.3390/s21175976

**Published:** 2021-09-06

**Authors:** Inês Soares, Marcelo Petry, António Paulo Moreira

**Affiliations:** 1 Department of Electrical and Computer Engineering, Faculdade de Engenharia da Universidade do Porto, FEUP, 4200-465 Porto, Portugal; ines.o.soares@inesctec.pt (I.S.); amoreira@fe.up.pt (A.P.M.); 2 INESC TEC—Institute for Systems and Computer Engineering, Technology and Science, 4200-465 Porto, Portugal

**Keywords:** augmented reality, collaborative robots, industrial robots, programming by demonstration

## Abstract

The world is living the fourth industrial revolution, marked by the increasing intelligence and automation of manufacturing systems. Nevertheless, there are types of tasks that are too complex or too expensive to be fully automated, it would be more efficient if the machines were able to work with the human, not only by sharing the same workspace but also as useful collaborators. A possible solution to that problem is on human–robot interaction systems, understanding the applications where they can be helpful to implement and what are the challenges they face. This work proposes the development of an industrial prototype of a human–machine interaction system through Augmented Reality, in which the objective is to enable an industrial operator without any programming experience to program a robot. The system itself is divided into two different parts: the tracking system, which records the operator’s hand movement, and the translator system, which writes the program to be sent to the robot that will execute the task. To demonstrate the concept, the user drew geometric figures, and the robot was able to replicate the operator’s path recorded.

## 1. Introduction

If we look back a few decades, the factories had big lines of operations where repetitive work was done by humans. Most of these workers had injuries or fatalities because they had to perform the same movement thousands of times a day. Now, this hazardous work is made by machines with high accuracy, which allows humans to conduct tasks that demand critical judgment [1].

The world is living the fourth industrial revolution, also known as Industry 4.0 [2]. An important aspect of this manufacturing paradigm is the increasing intelligence of manufacturing systems and decentrally connected cyber-physical systems. The term intelligence refers to adaptability, autonomy, and flexibility through the decentralized decision making and an increased data generation and processing [3]. The increasing need for automation in the industrial environment is due to the markets becoming more fast-moving and complex. This reduces the product’s life cycle and increases the product variety, particularly, being more relevant in assembly tasks [4].

Nevertheless, some tasks are too complex or too expensive to be fully automated. Therefore, it would be more efficient if the machines were able to work with the humans, not only by sharing the same workspace but also as useful collaborators [5]. A possible solution to that problem is through human–robot interaction systems, by understanding the applications where robots can be helpful and what are the challenges they might face. Collaborative robots can be an ally, working alongside humans. This enables the human to perform critical tasks that demand reasoning and reflection, leaving the repetitive and heavy ones to the robots. However, programming this collaborative robot can be a complex task that demands expertise in robot programming. For the manufacturing companies, it translates into extra costs, and the need to hire and train dedicated personnel.

However, is it possible to enable operators to program robots, even when they do not have any programming knowledge? In an attempt to solve that problem, Learning by Demonstration uses Machine Learning to program the robots through policies. This way, the human does not need to work on complex algorithms, the computer generates them by training the models. Conversely, this implies huge reliability on well-constructed datasets to achieve valid results and a relatively large amount of time to train the models [6].

As an alternative method, Augmented Reality (AR) can be a viable solution to explore to improve the effectiveness of tasks executed in the industry environment. In this sense, this work presents a prototype in which an operator, with no knowledge in robot programming, can program a collaborative or industrial robot. The system was implemented in Microsoft HoloLens 2, a head-mounted device that the operator could wear. AR technology is used to teach the robot what tasks it must perform, enabling the operator to program by demonstration.

The focus of this work is timely as it is necessary to learn and implement the technologies which most contribute to finding solutions for simpler and more efficient work in the industry. This way, the companies can save time and money finding a specialist to do it. Thereby answering the research question formulated: can an operator (without programming skills) program robots by demonstration using AR technology?

## 2. Related Work

Industry 4.0 is focused on improving productiveness and enhancing the user experience, which are key features of AR. Human–robot collaboration is one of the main application areas of AR in the industry at the moment [7,8]. The pandemic COVID-19 accelerated the evolution of AR and the worldwide spending on these technologies is expected to grow from $12.0 billion in 2020 to $72.8 billion in 2024 [9]. According to the International Data Corporation, the 5-year compound annual growth rate will be 54%.

Programming by demonstration can be a very important tool for an operator that does not have experience or knowledge of computer programming at all. This way they would be able to program the robot just by doing the task themselves and then the robot would do the same.

Aleotti et al. [10] proposed a visual-haptic AR system for manipulating objects and task learning from human demonstration. This proposal it is used a haptic device for object interaction and a desktop AR setup. The manipulators of the haptic device are located remotely, not in an environment where the real objects are presented. The object recognition and registration are performed automatically by a moving laser scanner mounted on a robot arm. The results obtained with the experiments performed show that the learned task can be successfully executed by the robot system.

Araiza-Illan et al. [11] proposed a system to re-program robot packing intuitively through simple hand gestures and information gathered by the AR device (HoloLens). The experiment setup was composed of a UR10 robot, a multi-finger suction gripper, a wrist Robotiq camera, two types of objects (sugar sachets, and coffee pods), two trays with distinct QR markers, and the HoloLens device. The wrist camera was added to increase the accuracy of the information acquired. In the AR interface, the operator matches each object to the corresponding tray with hand gestures, and the resulting pick-and-place program is sent to the robot. The robot was able to execute the task by placing the objects of a certain type on the respective tray and then repeating it for the other type of objects. If new objects were added after completion, the robot would continue the task until the objects were all organized. This way it was possible to quickly re-configure the packing application without having previous robot programming knowledge.

Rudorfer et al. [12] presented an intuitive drag-and-drop programming method using AR that could be performed by an operator without robot programming knowledge. In the implementation the devices used were the Microsoft HoloLens and the UR5 robot, integrated into a framework of web services. The main objective was for the user to pick a recognized object and place it in the desired location so that the robot could imitate it. The robot started by acquiring the image, then it recognized the object and its pose. After recognizing all the objects, the objects were displayed in the AR device, overlaying the real ones. Then, the robot control module extracted the initial and final coordinates of the desired locations and performed the referential transformations from the camera referential to the robot referential. Finally, the pick and place task could be executed. The results obtained by the prototype developed were successful but the robot’s accuracy was unsatisfactory.

Blankemeyer et al. [4] developed an AR application for HoloLens and the prime objective was to enable operators to program a pick-and-place task in an industrial robot by linking real and virtual objects. For that, the user had to move the virtual object to the desired position. Then the coordinates of the start and end points had to be transformed from the internal coordinate system of the HoloLens into the robot’s base coordinate system. Finally, the path planning was carried out directly by the robot controller. The results of the tests performed showed that the robot was able to complete the tasks with two components, but the researchers assured that the same could be expected when adding more components.

In the works referred to in the literature, the user moves virtual objects to the desired points, and the robot replicates the paths in the real world. Conversely, the purpose of this study is to develop a system where the operators can virtually draw with their hand the robot’s path. This approach avoids the usage of markers, providing smoother and easier path recording.

## 3. Materials and Methods

**Figure 1:**
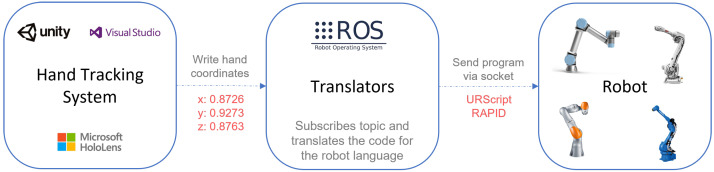
System’s overview.

The system developed to program robots using AR was divided into two different subsystems: the one where the path data were acquired and the one responsible for translating the hand coordinates to the robot’s language and sending them to the robot. The main objective of the first one was to provide a simple and smooth interface, which had to be intuitive and cannot contain any distractions, otherwise, the operator could be confused with the excessive information. The second one focused on the development of the translators that transformed the received hand coordinates into the different robot programming languages. As the robots used for the implementation were the Universal Robots UR5 and the ABB IRB 2600, the translators developed could generate code for the languages URScript and RAPID. It was opted to develop these translators in Robot Operating System (ROS). Figure 1 represents the system overview.

- Hand movements were captured by the Microsoft HoloLens 2 (HL2), using the platforms Unity and Visual Studio to program the application, and then the data were properly transmitted through a Robot Operating System (ROS) topic;
- In ROS, some nodes could subscribe the data received in the specific topic and translate the list of coordinates acquired to the robot language before sending to the robot;
- Finally, the ROS node connected to the Robot via a socket, and the generated program was sent to the robot, which executed the previously recorded movement.

### 3.1. Augmented Reality Based Robot Programming System

The interaction with the user was designed to be as simple as possible. So, the method chosen to perform it was to use popup windows. This way they always appeared in the middle of the user’s field of view and were not anchored in the same place like it would happen with buttons, which could induce some confusion in the user. Additionally, when these popup windows appear, they produced a sound, warning the user that the application state had changed.

#### 3.1.1. Workspace Setup

The interface was divided into three different parts; the first one was where the user chose what robot was going to be programmed, the second part was where the robot’s coordinate system was defined, and the third defined the robot workspace. For the purpose of this work, two different types of robots were chosen to test the developed system, a collaborative (Universal Robots UR5) and a traditional industrial robot (ABB IRB 2600). The objective was to integrate two different types of robots and demonstrate that the programming by demonstration methodology proposed could be generalized to any type of industrial manipulator, therefore, simplifying the operator’s work in the plant floor.

In order to differentiate the type of robot that the user wanted to program, when the tracking application was launched, a popup dialog showed up asking the user to choose the type of robot. This choice would influence the future configurations of the application, such as the robot workspace.

To be able to program the robot correctly, the robot’s and the HoloLens 2’s coordinate systems had to match. The HoloLens 2 coordinate system was defined when the application was initiated at a specific distance from the headset, and it was different every time the application was launched. In order to solve this problem, a manual coordinate system had to be define by the user. The HoloLens 2 software did not allow us to define a second coordinate system, so the method found to work around this problem was to place an object with the desired pose and then estimate the hand coordinates in relation to that object and not in relation to the headset. The object chosen was the Gizmo, which represented a three-axis referential and could verify if the coordinate system was correctly defined.

**Figure 2:**
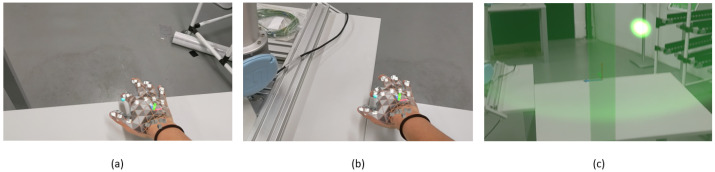
Coordinate system definition: (**a**) finger marking the origin’s place. (**b**) Finger marking the point that would define the referential frame orientation. (**c**) Setup finished.

The HoloLens 2 had an Inertial Measurement Unit (IMU) that defined the *Y*-axis always pointing upwards (despite the orientation in which the application was launched), enabling the coordinate system to be defined with only two points—the origin and the *Z*-axis orientation (Figure 2). To minimize the errors of both coordinate systems matching, it was relevant to have in mind that the points were defined by the projected hand hologram and not the real hand. Summing up, the application’s coordinate system was defined by placing the Gizmo in the position of the first point defined by the user and applying a rotation, so that the *Y*-axis was pointing forward. This way, the coordinate system defined in the interface was the one where the Y-Z plane matched the robot’s base plane and the *X*-axis pointing upwards.

A previous study [13] experimentally evaluated Hololens 2 hand tracking accuracy using OptiTrack motion capture system (a sub-millimetric precision system) as ground truth. The experiments were performed by different users to evaluate Hololens 2 behaviour with different hand sizes and shapes, as well as different hand movements and velocities. The results demonstrated an accuracy between 1 and 3 cm, which indicates that this system is suitable for applications that do not require high precision standards.

To prevent the operator to perform movements that could not be reproduced by the robot, an hologram of the robot’s workspace was projected into the environment. In addition to this visual assistance, the application also monitored the operator’s movement and warns him/her whenever the robot’s workspace was violated.

The robot workspace was adapted accordingly to which robot the user defined, namely, the dimensions of the objects that limited the working area. The workspace was projected when the user finalized the coordinate system definition and the object’s materials were green, only when the user was recording, and when the limits were violated, the materials turned red, returning to green after confirming in the Dialog that another recording had to be performed.

**Figure 3:**
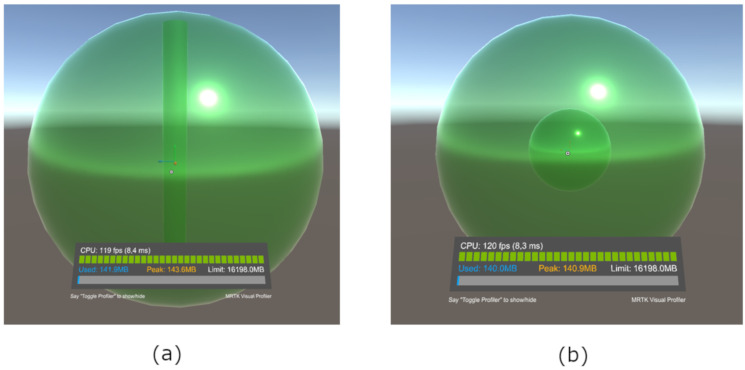
Projected workspace: (**a**) Universal Robots UR5. (**b**) ABB IRB 2600.

For the Universal Robots UR5, the robot’s workspace was quite straightforward to draw (Figure 3a). So a cylinder inside a sphere was used to create the workspace. The sphere had a diameter of 1.7 m, as it was the recommended reach, and the cylinder had a diameter of 0.151 m and a total height of 1.621 m. The available space to record the movement was the one which intersected the area outside the cylinder and inside the sphere.

The user exited the robot’s workspace when his/her right index finger tip has a distance from the *Gizmo* (calculated as in Equation (1)) higher than 0.85 m (radius of the sphere) or a distance from the Gizmo (calculated as in Equation (2)) lower than 0.0755 m (radius of the cylinder). The calculation to see if the user entered the cylinder was done only with the *x* and *z* measurements because, as the object was a cylinder, the height was limited with the sphere restriction.

(1) $$Sphere\text{\_}distance=\sqrt{x_{Gizmo}^{2}+y_{Gizmo}^{2}+z_{Gizmo}^{2}}$$

(2) $$Cylinder\text{\_}distance=\sqrt{x_{Gizmo}^{2}+z_{Gizmo}^{2}}$$

where, *x_Gizmo_*, *y_Gizmo_*, *z_Gizmo_* are the coordinates of the user’s right index finger tip in relation to the *Gizmo*.

The ABB IRB 2600 workspace was not as simple as UR5. In order to simplify the drawing of the 3D object representing the workspace, the robot’s workspace was approximated by two spheres, one inside the other, as represented in Figure 3b. The diameters of the spheres were set to 2.90 m for the maximum limit and 0.94 m for the minimum limit.

The method used to verify if the user was recording the movement within the robot’s workspace was similar to the one used for UR5, but, as in this case the workspace is constituted by two spheres, the equation used to calculate the maximum and minimum distance was Equation (1). It is worth urging the fact that this design was an approximation of the robot’s model, as the 3D object drawing was not the core of this work.

#### 3.1.2. Path Recording

This application required being as simple and flexible as possible. In that sense, the use of buttons was avoided because it could cause some confusion to the operator. Therefore, the use of gestures was preferred. For convenience, the Air Tap [14] gesture was chosen to start and finish the path recording. This movement begins with the hand opened, then touching the thumb with the index finger and, finally, pointing the index finger straight up toward the ceiling again. The algorithm could recognize Air Tap gestures from both hands (right and left), so the user could choose the preferred one.

**Figure 4:**
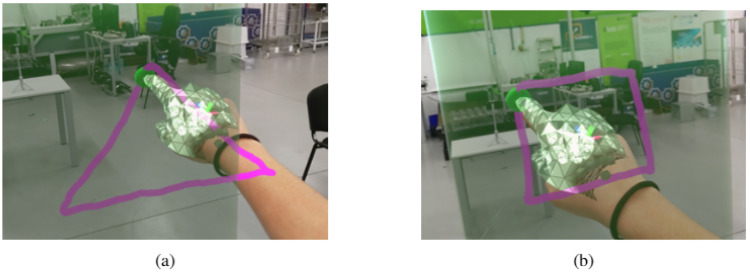
Example of recorded paths: (**a**) triangle. (**b**) Square.

In an effort to simplify the user’s awareness of the system’s state, a small sphere was added in the right index finger tip; and it was red when the system was not recording and turned green while it was recording. Additionally, to increase the perception of the movement recorded, while the user performed the movement the systems drew a line representing the path. This way, when the user finished the recording, he/she could verify if the movement was done correctly or if it was not and needs to be repeated. To contrast with typical materials found in industrial settings, the recorded path is represented in hot pink. The Figure 4 depicts examples of two paths recorded.

The coordinates to send to the robot are the ones relative to the *Gizmo* object, representing the robot’s origin. The function developed to perform the transformation had two major steps: the first one was to calculate the distance vector between the absolute coordinate (point2transform) and the *Gizmo* position ($origin_{Gizmo\_frame}$)—Equation (3), and the second one was to calculate the relative position (relative_coordinates), as shown in Equation (4), by the product of the rotation transformation matrix ($R_{Gizmo\_frame}$) of the Gizmo’s referential frame and the vector_distance, plus the Gizmo’s translation vector ($T_{Gizmo\_frame}$). It is worth mentioning that the HoloLens 2 coordinate system was left-handed, whereas the one used in the robot was right-handed. So, one of the axes was inverted by multiplying it by −1; this way the application referential resulted in a right-handed one.

(3) $$vector\_distance=point2transform-origin_{Gizmo\_frame}$$

(4) $$relative\_coordinates=R_{Gizmo\_frame}\cdot vector\_distance+T_{Gizmo\_frame}$$

After collecting the path coordinates, to increase the level of abstraction and compatibility, they were sent to Robot Operating System (ROS), since ROS drivers already exist for several robot models. Thereafter the data were analyzed and a program built to send to the robot. In order to facilitate the communication between the HoloLens 2 application and ROS, it was used as base the ROS# library [15].

### 3.2. Program Translators

**Figure 5:**
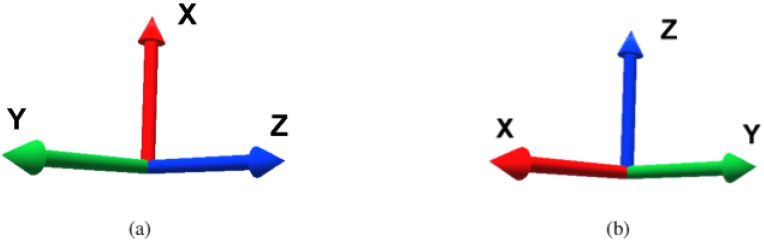
Coordinate system comparison: (**a**) AR application coordinate system. (**b**) Robots’ coordinate system.

A ROS package was created to implement the two program translators, one for each robot programming language required (URScript and RAPID). There was one additional adjustment to be done to the coordinates, because in HoloLens 2 application the coordinate system was defined to have the X axis pointing upwards, whereas the robots’ coordinate system had the Z axis pointing upwards. Thereby, the coordinates were matched as shown in Equation (5), where a rotation matrix was applied between both referentials (Figure 5 represents that matching graphically).

(5) $$\left[\begin{array}{c} x_{robot}\\ y_{robot}\\ z_{robot} \end{array}\right]=\left[\begin{array}{ccc} 0 & 1 & 0\\ 0 & 0 & 1\\ 1 & 0 & 0 \end{array}\right]\left[\begin{array}{c} x_{HoloLens}\\ y_{HoloLens}\\ z_{HoloLens} \end{array}\right]$$

There were three major steps that the C++ programs followed to translate the coordinates into the robot programming language. First, when the ROS topic received a signal indicating that the coordinates list was going to be transmitted, the robot program was initialized. Then, when the topic started receiving the hand coordinates recorded, they were added to the robot program with the adjustments explained previously (Equation (5)). Finally, when the topic received a signal indicating the end of the transmission, the robot program was finalized and closed. This procedure had some minor differences according to the robot that was programming, which will be explained in the following subsections.

#### 3.2.1. Universal Robots UR5

Firstly, to demonstrate the concept of this work, the program was developed for the Universal Robots UR5. It was possible to control this robot through different levels, namely, the Graphical User-Interface Level, the Script Level and the C-API Level [16]. The method chosen was the Script programming because it would enable the connection between ROS and the UR5 controller through a TCP/IP socket.

As the desired process was to move the robot’s end-effector to the received coordinates, replicating the user’s hand movements, the function used in the URScript program was *movel*. This function moved the end-effector to the specified position linearly in tool-space, and it took the following variables as arguments:

- Target pose, which was constituted by the x, y and z coordinates in meters and the rotations in those axis (rx, ry and rz).
- Tool acceleration, in meters per squared seconds.
- Tool speed, in meters per second.
- Time, which is movements duration and is represented in seconds.
- Blend radius, which was the tolerance in which the robot’s control assumed that the end-effector reached its location, and is represented in meters.

When using this function, the programmer could either choose to define the end-effector’s velocity or the time in which the movement must be executed. In this case, it was opted to use the velocity instead of the time. All the parameters’ values could be adapted for the application in which the robot was going to work, this way the robot’s movement could be adjusted and its speed increased or decreased accordingly. Table 1 shows an example of a program generated by the ROS nodes with the coordinates recorded by the AR application to program UR5, using the programming language URScript.

#### 3.2.2. ABB IRB 2600

The industrial robot ABB IRB 2600, as it is not a collaborative robot, it did not have strength and pressure safety sensors. One of the critical issues was controlling the robot’s speed, as it was much faster than a collaborative robot. The controller mode of the robot was defined as semi-manual, meaning that the speed would be automatically reduced to half and the robot would only move when the user was pressing a button on its teaching pendant; the moment the user let loose the button, the robot would stop. Additionally, when executing the program, in the teaching pendant, the speed was defined to be 50%, consequently, in total the speed was 25% of its programmed value. These security measures were necessary for the testing steps, but when the programs are correctly verified, the robot’s velocity can be increased accordingly to the application requirements.

The language used to program this robot was RAPID, as it is a high-level programming language used to control ABB industrial robots. The generated file that contained the program had to be initialized, declaring the module and invoking the main function and then finalized.

The function used to move the robot to the desired positions was *MoveL*, which moved the tool center point linearly to a given target, and it took as arguments the variables shown below [17].

- ToPoint, which had the data type robtarget, and provided the target point of the robot and the external axis. This variable was defined by four different arrays:
  1. The x, y and z vector, which represent the robot’s target position in millimeters, from the recorded movement by the HoloLens 2 application.
  2. The quaternion q1, q2, q3 an q4, which represented the orientation.
  3. The robot configuration for axis 1, 4, 6 and external.
  4. The configuration of the external joints angles, it was possible to control six external joints by default.
- Speed, which represented the velocity of the tool center point in millimeter per second. Alternatively to the speed input, it can be also specified the time in which the robot should move.
- Zone, which defined the accuracy in millimeters of the robot’s tool center point.
- Tool, which specified the tool in use when the robot moved, the tool center point was then moved to the target position.

## 4. Results

**Figure 6:**
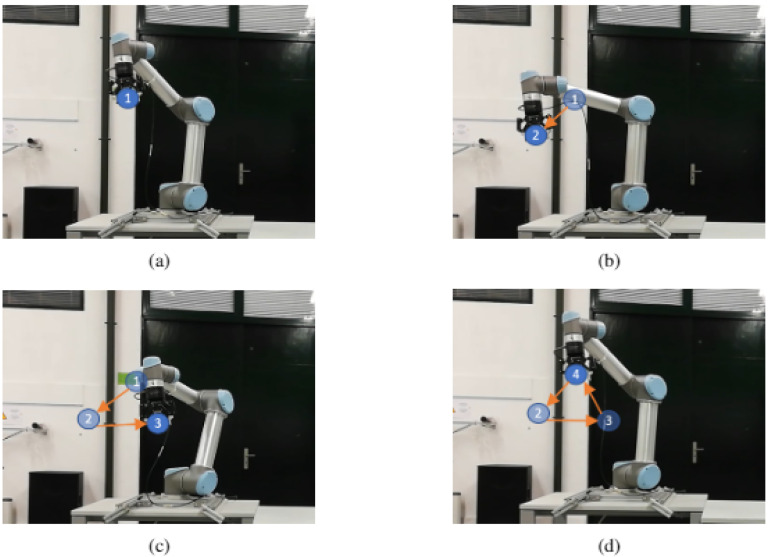
UR5 path execution (triangle): (**a**) Robot at initial point. (**b**) Robot at second point. (**c**) Robot at third point. (**d**) Robot at final point.

**Figure 7:**
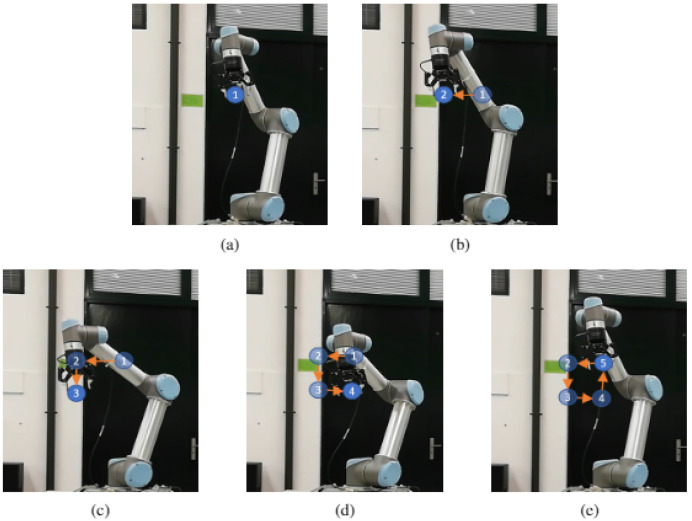
UR5 path execution (square): (**a**) robot at initial point. (**b**) Robot at second point. (**c**) Robot at third point. (**d**) Robot at fourth point. (**e**) Robot at final point.

In the following link (https://youtu.be/joV-4uArWDw, accessed on 18 August 2021) a video is displayed illustrating the experiments performed. Although any path could be programmed, the first experiment on UR5 was drawing a triangle, for being easy to detect if it was being correctly performed (three vertices and three edges). Figure 6a,d show the different moments where the robot reached a vertex. The blue circles numbered and the orange arrows were added to mark the previous vertices reached by the robot, for a easier understanding of the movement through the images. Additionally, another experiment was performed: a square. Figure 7 shows the different moments where the robot reached each vertex (Figure 7a,e).

**Figure 8:**
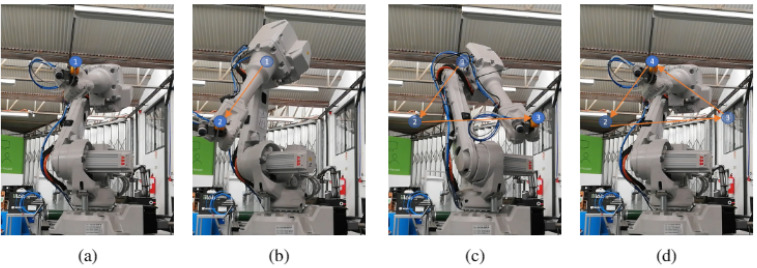
ABB IRB 2600 path execution (triangle): (**a**) Robot at initial point. (**b**) Robot at second point. (**c**) Robot at third point. (**d**) Robot at final point.

**Figure 9:**
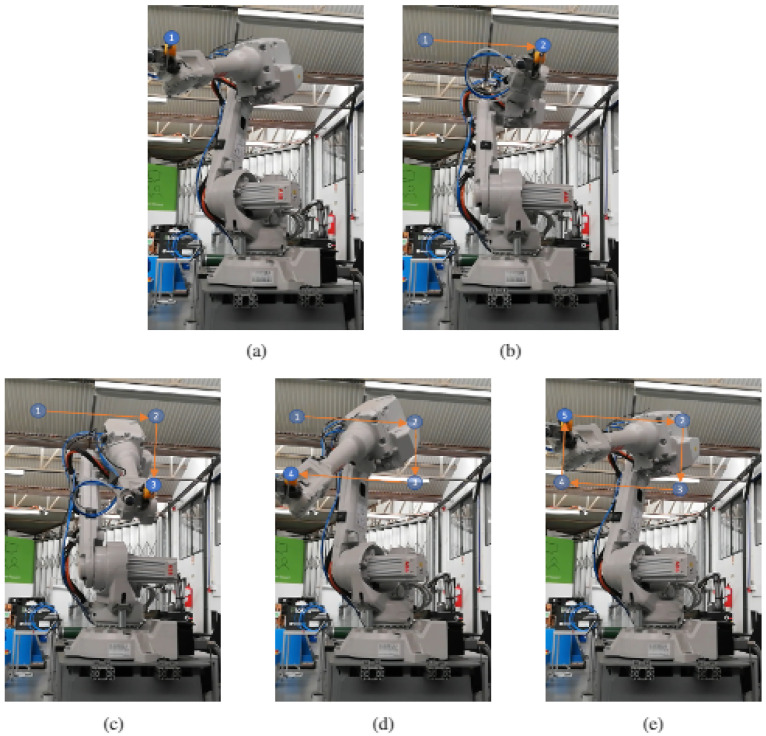
ABB IRB 2600 path execution (rectangle): (**a**) Robot at initial point. (**b**) Robot at second point. (**c**) Robot at third point. (**d**) Robot at fourth point. (**e**) Robot at final point.

The first experiment on ABB IRB 2600 was drawing a triangle, for being easy to detect if it was being correctly performed (three vertices and three edges). Figure 8 shows the different moments where the robot reached a vertex. The blue circles numbered and the orange arrows were added to mark the previous vertices reached by the robot, for an easier understanding of the movement through the images. Additionally, another experiment was performed: a rectangle. Figure 9 shows the different moments where the robot reached each vertex.

**Figure 10:**
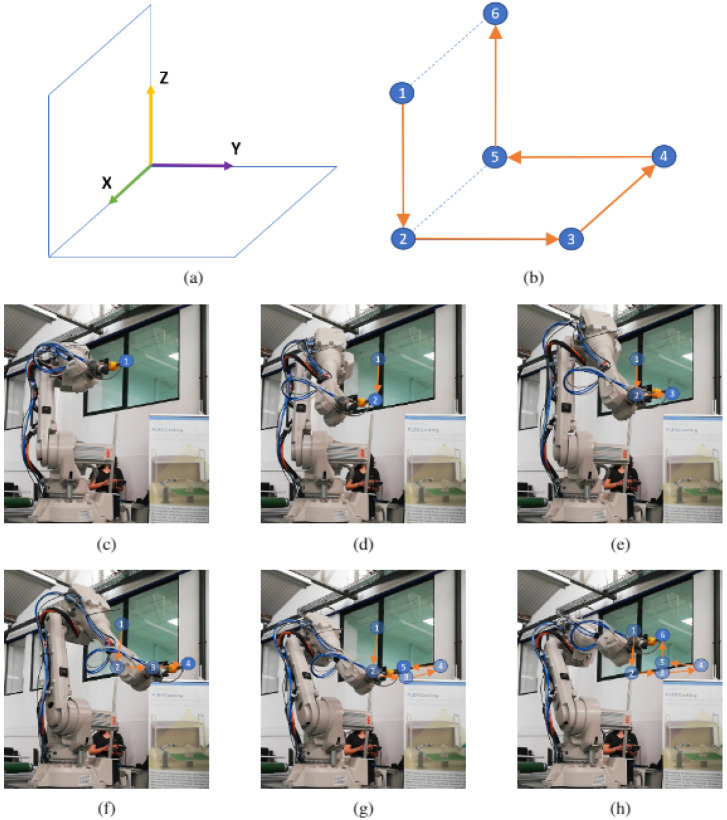
ABB IRB 2600 path execution (3D solid): (**a**) path referential frame. (**b**) Path sequence. (**c**) Robot at initial point. (**d**) Robot at second point. (**e**) Robot at third point. (**f**) Robot at fourth point. (**g**) Robot at fifth point. (**h**) Robot at final point.

A third experiment was performed in a three-dimensional space. Figure 10a shows the different directions of the drawing following the three axis. In that sense, a three-dimensional figure was drawn like the Figure 10b. The robot started in point 1 and moveed through the *Z* axis to point 2; then, through the *Y* axis reached point 3; next, to go to point 4 moved along the *X* axis; afterwards, to go to point 5 advanced through the *Y* axis; finally, to go to point 6 proceeded through the *Z* axis. Figure 10c,h try to show that three-dimensional drawing.

## 5. Discussion

The main objective of these experiments was to demonstrate the concept of programming a robot by demonstration using AR. After analysing the results of these five experiments, it is possible to conclude that the goal was achieved with both robots. It was chosen to use robots from different brands to exhibit the feasible generalization. Nevertheless, there is always room for improvement. In that sense, this section intends to enumerate possible improvements to enhance the developed product for a better user experience and simplicity.

The first simple improvement that could be implemented is the rate of the coordinates recording. At this point the programs records the coordinates at every frame, but it could be simplified to record at a fixed rate and a more far-between sample acquisition. As a matter of fact, this sampling rate could even be adjusted accordingly to the application in which the system will be applied.

One possible upgrade could be to project an arrow in the headset indicating the next robot’s movement. This way the operator would be able to anticipate possible reactions and feel more comfortable alongside the robot.

A possible addition that can be integrated is to consider the hand orientation when recording. As it was explained, for this case it was only considered the position, so the usage of the hand’s orientation would make the range of possible applications of the product wider. For example, enabling it for pick and place applications where some objects require specific movements to be executed. Following that example of a pick and place application, it would also be a great addition the possibility of controlling the robot’s end-effector.

Furthermore, the possibility of cutting out the intermediary of the whole process, ROS in this case, would simplify the user experience for an operator without any programming knowledge. To accomplish that, the translators would have to be implemented in the AR application along with the sockets to communicate with the robots. These additions could slightly slow down the application, but the advantages that it would provide are more significant. The main advantage is the fact that the system can work only with the HoloLens 2 and the robots, avoiding the use of an external computer.

## 6. Conclusions

The prime objective of this work was to develop a system that would provide the operator without coding knowledge, a way to program robots using AR technologies. Therefore, this work proposed a case study where the operator would use a HoloLens 2 device to record movements, and then the robot would replicate those paths.

The system was divided into two different parts: the AR application that would record the operator’s hand movements, and then the translators that would transform the recorded path coordinates to the robot language required.

The AR application had the primary goal of providing the user with a simplistic and easy-to-use application without distractions. In that sense, the interaction with the interface is made by hand gestures and popup windows, avoiding the use of buttons that could confine the user to a specific zone. With the coordinate system definition done by the user, the application projects the robot workspace according to its specifications, which prevents the operator to record movements out of the robot’s reach.

In order to test the developed system and to validate it, some experiments were made to demonstrate the concept proposed initially. In that sense, it was opted to draw geometric figures, because it would be easy to verify if the robot was able to replicate the path. Therefore, the chosen paths were a triangle, square/rectangle, and a three-dimensional solid to test that the path drawing could be done in a 3D space. These experiments were considered successful in both robots, as they were able to replicate the recorded path. It is worth mention that the orientation and velocity imposed in these experiments were always the same, although that can be altered accordingly to each robot’s application. Additionally, it was also tested the case where the user would try to record a path outside the robot’s workspace, which was immediately interrupted and the user had to restart the recording.

The obtained results allow us to demonstrate the concept of this work, thereby answering the research question formulated: enabling an operator to program a robot by demonstration using AR technology. This work used two robots, a collaborative and an industrial one, nevertheless the application was built to easily add new ones. For that, the programmer only needs to insert the robot’s parameters to define its workspace, and build the correspondent translator. If this work was to be integrated into Industry, some remarks had to be considered regarding the robot’s path execution. Namely, the robot’s speed and blend radius that was hard-coded for these experiments. Accordingly to the robot’s application, the speed can be increased and the blend radius adjusted to the desired accuracy of the path execution.

In conclusion, this work provides a viable, effortless, and economic solution for programming robots by demonstration, with no need for programming skills. The core of the work is to avoid the use of markers, providing a smoother and easier path recording method. Moreover, this approach is computationally light-weighted compared to other Learning from Demonstration techniques, such as Machine Learning approaches. Although, it is worth mentioning that, due to the system’s accuracy, the proposed work can only be implemented in applications that do not require less than one-centimeter error.

## Figures and Tables

**Table 1:** URScript program example for UR5.

def myProg():
movel( p [ 0.324881, 0.118973, 0.253622, 2.2, 2.2, −0.3 ], a = 0.01, v = 0.5, r = 0.1)
movel( p [ 0.324776, 0.117087, 0.252939, 2.2, 2.2, −0.3 ], a = 0.01, v = 0.5, r = 0.1)
movel( p [ 0.324623, 0.115607, 0.252753, 2.2, 2.2, −0.3 ], a = 0.01, v = 0.5, r = 0.1)
movel( p [ 0.324372, 0.113685, 0.252663, 2.2, 2.2, −0.3 ], a = 0.01, v = 0.5, r = 0.1)
end

## Data Availability

All data are contained within the manuscript. Raw data are available from the corresponding author upon request.

## Abbreviations

The following abbreviations are used in this manuscript:

AR	Augmented Reality
HL2	HoloLens 2
IMU	Inertial Measurement Unit
ROS	Robot Operating System
